# Insulin Therapy is Associated With Increased Myocardial Interstitial Fibrosis and Cardiomyocyte Apoptosis in a Rodent Model of Experimental Diabetes

**DOI:** 10.3389/fphys.2022.890907

**Published:** 2022-04-27

**Authors:** Fadi W. Adel, Ye Zheng, Siu-Hin Wan, Christie Greason, Shuchong Pan, Syed Ameenuddin, Horng H. Chen

**Affiliations:** ^1^ Cardiorenal Research Laboratory, Department of Cardiovascular Diseases, Mayo Clinic, Rochester, MN, United States; ^2^ Minneapolis Heart Institute, United Hospital, Saint Paul, MN, United States; ^3^ College of Biological Sciences, University of Minnesota, Minneapolis, MN, United States

**Keywords:** insulin, diabetes, fibrosis, apoptosis, rat model

## Abstract

The incidence of diabetes mellitus (DM) is rising. DM is a risk factor for developing left ventricular (LV) dysfunction and adverse cardiovascular outcomes. Insulin, commonly used to treat DM, is associated with further worsening of such outcomes. Yet, the pathophysiology of the adverse properties of insulin on the heart remains poorly defined. Therefore, the objective of this study was to determine the biological effects of insulin on the heart in DM, which we tested *in vivo* in a diabetic rat model and *in vitro* on human cardiomyocytes and fibroblasts. Male Wistar rats were divided into 3 groups: controls (*n* = 17), untreated diabetics (UDM, *n* = 15), and insulin-treated diabetics (IDM, *n* = 9). Diabetes was induced with Streptozotocin. Insulin pumps in IDM and saline pumps in UDM and controls were implanted for 4 weeks before tissue collection. Separately, cultures of human cardiomyocytes (AC16) and human cardiac fibroblasts (HCF) were treated with insulin to assess apoptosis and fibrosis, respectively. In rats, insulin partially rescued the DM-associated weight loss while fully restoring euglycemia. However, IDM had 2 × the rate of LV fibrosis (*p* < 0.0001) compared to UDM, and triple the rate of cardiomyocyte apoptosis compared to controls (*p* < 0.05). Similarly, *in vitro*, insulin triggered apoptosis in a dose-dependent fashion in AC16 cells, and it increased fibrosis and upregulated SMAD2 in HCF to levels comparable to Transforming Growth Factor Beta 1. Therefore, we conclude that insulin therapy is associated with increased cardiomyocyte apoptosis and myocardial interstitial fibrosis. Longer studies are needed to explore the long-term effects of insulin on cardiac structure and function.

## 1 Introduction

The incidence of diabetes mellitus (DM) has been on the rise in the United States and worldwide. In 2018, 10.5% of the United States population was diagnosed with diabetes, with an estimated 21.4% of whom being unaware of the diagnosis. Diabetes was the 7th underlying cause of death in the United States (Prevention and C.f.D.C.a, 2020). Worldwide, the prevalence of diabetes among adults increased from 4.7 to 8.5% between 1980 and 2014 (Organization and W.H, 2016).

DM is a known risk factor for the development of ischemic heart disease. However, independent of coexisting ischemic heart disease or hypertension, diabetics are twice as likely as their non-diabetic counterparts to develop left ventricular (LV) dysfunction and congestive heart failure (CHF) (Nichols et al., 2004; de Simone et al., 2010). The phenomenon of left ventricular dysfunction in the absence of hypertension and ischemic heart disease is known as diabetic cardiomyopathy (Boudina and Abel, 2007).

Indeed, studies have demonstrated that diabetes was independently associated with elevated LV diastolic pressures (Klajda et al., 2020), a higher prevalence and incidence of CHF (Kannel et al., 1974; Nichols et al., 2004), and an increased risk of heart failure hospitalization (Kristensen et al., 2016). More importantly, despite addressing the DM-associated hyperglycemia, some glucose lowering agents used to treat DM are associated with higher rates of adverse cardiovascular outcomes, including insulin (Action to Control Cardiovascular Risk, 2008; McMurray et al., 2014).

Insulin use, which is common in up to one-third of DM patients (Trief et al., 2016), is an independent risk factor for the development of heart failure (Nichols et al., 2004; Wang et al., 2015) and LV dysfunction (Hirose et al., 2021). Moreover, among patients with systolic heart failure, insulin therapy was associated with a higher 1-year mortality rate (Smooke et al., 2005). Hypotheses to explain the worse outcomes associated with insulin include that insulin induces sodium-retention, and that its use can be indicative of a more advanced diabetic state (Cosmi et al., 2018). Yet, it remains unknown whether insulin has direct deleterious effects on the myocardium.

Previous studies have shown that insulin possesses profibrotic effects in non-cardiac tissues, such as adipose tissue (Hasegawa et al., 2018), as well as in stellate cells in both the liver (Cai et al., 2017) and the pancreas (Yang et al., 2016). Yet, whether insulin itself exerts any profibrotic effects on the left ventricle in diabetes remains unknown.

Hence, the objective of the current study was to investigate the direct actions of insulin on the myocardium in diabetes, both *in vivo* and *in vitro*. We hypothesized that insulin induces direct profibrotic effects on the left ventricle. Through a series of *in vivo* and *in vitro* studies, we were able to test this hypothesis.

## 2 Materials and Methods

### 2.1 Rat Model and Diabetes Induction

**FIGURE 1:**
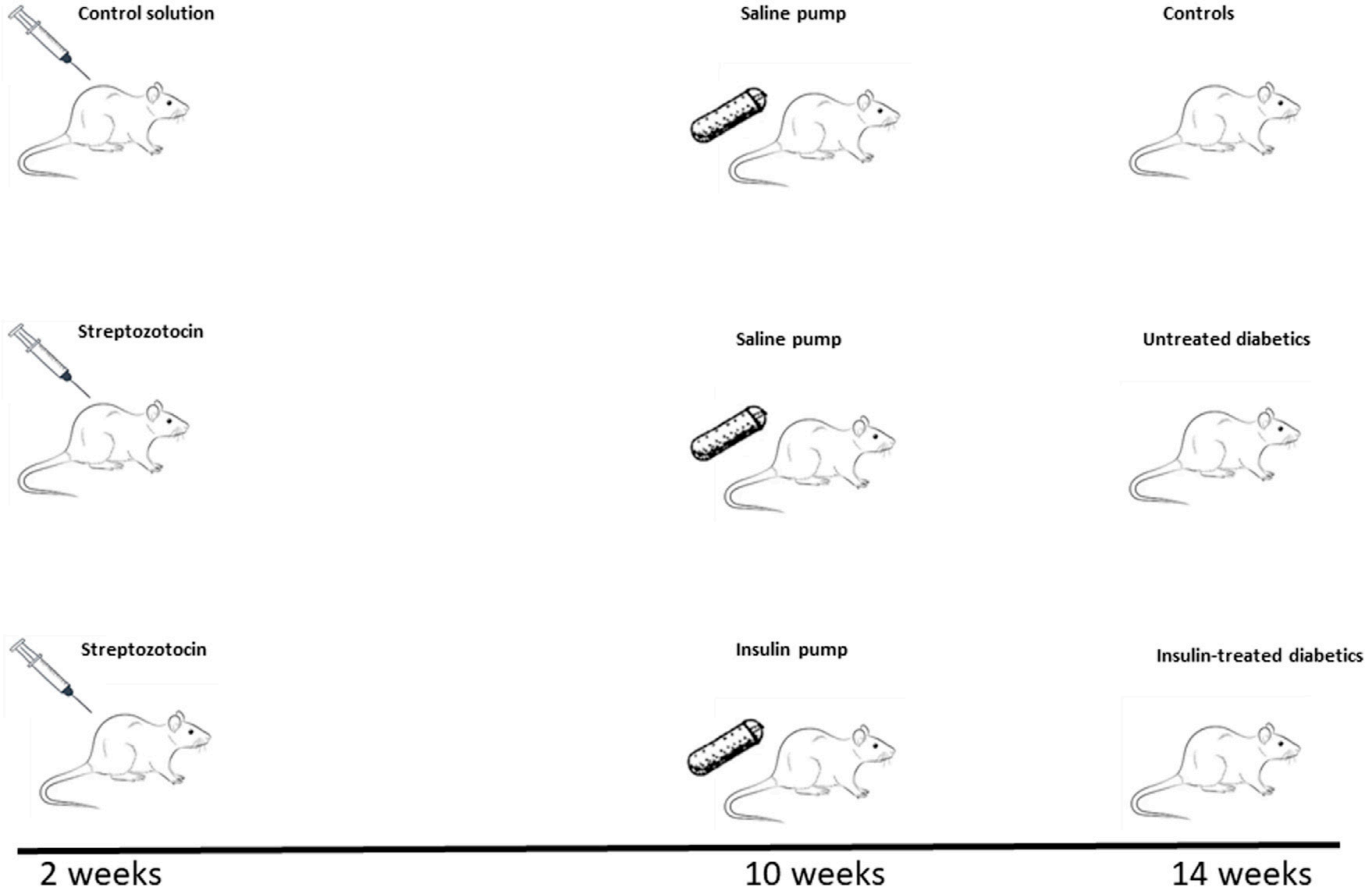
Study Timeline. After being maintained on a regular diet for 2 weeks, 4–6-week-old male Wistar rats were divided into three groups: 2 groups were injected with Streptozotocin (65 mg/kg) to induce diabetes and 1 group received a control solution. Eight weeks after the induction of diabetes (10 weeks of study time), saline-delivering pumps were implanted in the control and one of the diabetic groups (untreated diabetics, UDM), while insulin-delivering pumps were installed in the other diabetic group (insulin-treated diabetics, IDM). Therefore, at the end of the experiment at 14 weeks, three cohorts were inspected: controls (top panel), untreated diabetics (UDM, middle panel), and insulin-treated diabetics (IDM, bottom panel).

The Mayo Clinic Institutional Animal Care and Use Committee approved the study. Wistar rats (4–6 weeks old, male, Charles River Laboratories) were divided into 3 groups: controls (*n* = 17), untreated diabetic rats (UDM, *n* = 15), and insulin-treated diabetic rats (IDM, *n* = 9) (Figure 1). Rats were maintained on a normal diet throughout the 14-week study period. At 2 weeks, UDM and IDM groups received 65 mg/kg Streptozotocin (Sigma, St. Louis, MO) intraperitoneal injection. At 6 weeks, blood glucose was measured using a glucometer (OneTouch Ultra, LifeScan, Milpitas, CA). At 10 weeks, saline pumps (Alzet Osmotic Pump Model 2ML2, Cupertino, CA; infusion rate: 5.0 μl/h) were implanted in controls and UDM, and insulin (Humulin R, USP, rDNA origin, 100 IU/ml, Eli Lilly, Indianapolis, IN) pumps were implanted in IDM rats.

At 14 weeks, echocardiography and glucose analysis were repeated. Then, the rats were anesthetized (1.5% isoflurane) and placed on a heated temperature-controlled surgical table. The internal carotid artery and jugular vein were cannulated. After equilibration (60 min), blood was collected from the carotid artery. The collected blood in EDTA tubes/heparinized tubes was placed on ice and immediately centrifuged at 2,500 rpm at 4°C for 10 min. The plasma was stored in polystyrene tubes at−80°C. The hearts were harvested and weighed. The LV was dissected and split into two samples: one that was quickly frozen in liquid nitrogen and one that was preserved in 10% formalin for histological analysis. Fixed rat LV tissue was dehydrated, embedded in paraffin, and sectioned at a thickness of 4 μm. Picrosirius red staining was performed on sectioned tissues for the determination of collagen content and the extent of fibrosis using an Axioplan II KS 400 microscope (Carl Zeiss, Inc.). At least 4 randomly selected images were captured from each slide at × 20 magnification; KS 400 software was used to determine fibrotic area as a percentage of total tissue area.

### 2.2 Echocardiography

Standard transthoracic echocardiography was performed on anesthetized rats (1.5% isoflurane with 1 L oxygen) using the Vivid 7 ultrasound system (GE Medical Systems) and a 10S transducer (11.5 MHz) with electrocardiographic monitoring. M-mode images and gray scale 2D parasternal short axis images (300–350 frames/sec) at the midpapillary level were recorded for off-line analysis using EchoPAC software (EchoPAC PC BTO 9.0.0, GE Healthcare, Milwaukee, WI). LV internal diameter end diastole and end systole and wall thicknesses were measured from M-mode images permitting calculation of LV ejection fraction (LVEF) based on the cubed method and LV mass index. All parameters represented the average of 3 beats.

### 2.3 α-Smooth Muscle Actin Immunofluorescence Staining

Using tissue cultures of human cardiac fibroblasts (HCF) (PromoCell), myofibroblast expression of α-SMA was assessed. HCF were maintained in Fibroblast Growth Media 3 (PromoCell, Germany). HCF were serum starved for 48 h before either insulin (Sigma, 10 nmol/L), transforming growth factor beta 1 (TGFβ1) (R&D Systems, at 5 ng/ml), or both were added to the growth media of HCF for 72 h at 37°C. TGFβ1 was used as a positive control for inducing fibrosis. We chose 72 h since α-SMA expression by TGFβ1 peaks at 48 h and remains sustained at 120 h, and we wanted to ensure peak effect with both TGFβ1 and insulin (Thannickal et al., 2003). We chose insulin at a concentration of 10 nmol/L since it is equivalent to the dose of Humulin R U-100 used in treating diabetic humans (Lilly USA, 2011).

Subsequently, the media was removed, and cells were washed with phosphate-buffered saline (PBS) × 3. Cells were then fixed with 4% formaldehyde solution at room temperature for 20 min, and they were permeabilized with 0.2% Triton X-100 in PBS for 6 min on ice. Cells were then incubated with monoclonal anti-α-SMA antibody (1:200, Novus) at 4°C overnight. Subsequently, they were washed with PBS and incubated with a second fluorescent goat anti-mouse IgG antibody (1:500, Abcam) for 1 h at room temperature. Cells were then washed with PBS and mounted with 4′, 6-diamidino-2-phenylindole (DAPI, Vector Laboratories Inc). Fluorescent microscopy was performed under Nikon A5OR confocal microscopy. The expression level of α-SMA was measured by ImageJ software.

### 2.4 Collagen Secretion Assay

Collagen content in cell culture media was measured by Sirius Red Collagen Detection Kit (Chondrex, Inc) according to the manufacture’s protocol. Briefly, HCF were serum-starved for 48 h before being treated with insulin (10 nmol/L) or TGFβ1 (5 ng/ml) for 48 h (Petrov et al., 2002). Media was collected and briefly centrifuged (1,000 g for 5 min) to remove cell debris. Next, 500 µl of Sirius Red Solution was added to 100 µl of sample media and incubated at room temperature for 20 min. The collagen pellet was washed with Washing Buffer and subsequently dissolved in Extraction Buffer. Next, the solution’s optical density was quantified with a spectrophotometer (Molecular Devices) at 510 nm. All experiments were repeated three times. At least 4 random fields were selected from each slide. The areas represent the free wall of the mid left ventricle.

### 2.5 Cardiomyocyte Real-Time Apoptosis Analysis

Human cardiomyocyte cell line (AC16) was purchased from Millipore and maintained in DMEM/F12 media (Sigma) containing 2 mmol/L L-glutamine and 10% FBS. Cells were seeded in a 96-well plate and grown to approximately 80% confluency before experiments. They were then treated with insulin (10 nmol/L)- or TGFβ1 (5 ng/ml)- containing 1 × Essen Bioscience IncuCyte Caspase-3/7 Green Reagent (final concentration 5 μmol/L). Plates were then transferred to a time-lapsed, live imaging system, IncuCyte (Essen BioScience, Ann Arbor, MI), for apoptosis monitoring for a total of 48 h. All treatment groups were carried out in triplicates and repeated twice. Data from treated plates were collected with IncuCyte Zoom Live-Cell Analysis System. Images were collected every 2 h and analyzed for the number of apoptotic bodies per well.

### 2.6 Terminal Deoxynucleotidyl Transferase dUTP Nick End Labeling Assay

Apoptosis in rat LV tissue was performed on tissue sections (*n* = 7 for each group) using DeadEnd Flurometric TUNEL System (Promega) following manufacturer’s protocol. Briefly, paraffin was removed from tissue sections. Then, tissues were permeabilized with Proteinase K. Next, tissues were incubated with terminal deoxynucleotidyl transferase (TdT) reaction buffer for 60 min at 37°C. After washing the tissue with PBS, slides were mounted with mounting media containing DAPI. Apoptotic cells were detected as green, fluorescent bodies by confocal fluorescence microscopy (Nikon A50R, Melvile, NY). In each slide, 10 fields were randomly selected, and the number of green apoptotic cells and total cells were recorded. The apoptotic index was defined as the number of green apoptotic cells divided by the total number of cells per field.

### 2.7 Cell Culture and Western Blotting

Human cardiac fibroblasts were grown to 80% confluence in 100-mm tissue culture dishes and treated with insulin (10 nmol/L) or TGFβ1 (5 ng/ml) for 24 h. Cells were harvested in lysis buffer and homogenized to achieve a homogeneous solution. Sample protein concentrations were determined by the Bradford technique (Bio-Rad). To determine SMAD2 protein levels in whole cell lysates, 20 μg of total protein were size-fractionated by 12% SDS-PAGE and transferred to Polyvinylidene fluoride or polyvinylidene difluoride (PVDF) membranes. For Western blot analysis, membranes were incubated in a blocking solution containing 5% dry milk in PBS + Tween 20 for 1 h, followed by overnight incubation with rabbit anti-SMAD2 antibodies (1:500, Abcam) or rabbit anti-Glyceraldehyde-3-Phosphate Dehydrogenase (GAPDH) antibodies (1:2,000, Abcam; Cambridge, MA). The membranes were then incubated for 1 h with horseradish peroxidase-conjugated anti-rabbit IgG antibody (1:5,000, Calbiochem; San Diego, CA) and visualized using chemiluminescence detection by Chemi Doc (Bio-Rad, CA). The results of the Western blots were analyzed using ImageJ.

### 2.8 Statistical Analysis

Continuous variables were reported using mean ± standard error of the mean (SEM) if normally distributed or median (25th percentile, 75th percentile) if otherwise. GraphPad Prism 9 (San Diego, CA) was used for statistical analysis. ANOVA or Kruskal-Wallis tests were used to compare all three groups and, if statistically significant, pairwise Student’s *t*-tests or Mann Whitney U tests were performed, respectively. Graphs were produced using Microsoft Excel 365. All hypothesis tests were 2-sided, and a *p*-value of < 0.05 was considered statistically significant.

## 3 Results

### 3.1 *In Vivo* Rat Studies

#### 3.1.1 Baseline Characteristics

A total of 41 male Wistar rats were divided into three groups: controls (*n* = 17), untreated diabetic rats (UDM, *n* = 15), and insulin-treated diabetic rats (IDM, *n* = 9). Diabetes was induced in both UDM and IDM using intraperitoneal Streptozotocin injections at week 2. At week 6, hyperglycemia was confirmed in UDM (26.75 ± 0.66 mmol/L) and IDM (25.12 ± 1.07 mmol/L). At week 10, insulin pumps in IDM and saline pumps in UDM and controls were installed for 4 weeks.

At the time of the study, which was at 14 weeks (Figure 1), insulin therapy partially rescued the diabetes-associated weight loss observed in untreated diabetic rats (Table 1). IDM rats achieved euglycemia at levels similar to controls (6.05 ± 0.88 vs. 5.93 ± 0.17 mmol/L, *p* = 0.8651) while hyperglycemia was maintained in UDM (24.61 ± 1.10 mmol/L) (Table 1).

**TABLE 1:** Characteristics and Cardiac Parameters at 14 weeks.

	Control (*n* = 17)	UDM (*n* = 15)	IDM (*n* = 9)	*p*-value: Control vs. UDM	*p*-value:UDMvs. IDM	*p*-value: Control vs. IDM	*p*-value:All groups
Characteristics and Cardiac Parameters							
Body weight (g)^a^	526.0 (494.0,555.0)	354.0 (334.0,396.0)	434.0 (416.0,463.0)	<0.0001	0.0023	0.0002	<0.0001^b^
Blood glucose (mmol/L)^c^	5.93 ± 0.17	24.61 ± 1.10	6.05 ± 0.88	<0.0001	<0.0001	0.8652	<0.0001^d^
MAP (mmHg)^c^	101.10 ± 2.25	97.31 ± 3.81	92.69 ± 3.33	—	—	—	0.2292^d^
HR (bpm)^c^	370.8 ± 7.36	327.2 ± 7.41	301.3 ± 7.88	0.0002	0.0331	<0.0001	<0.0001^d^
LVEF (%)^c^	81.06 ± 1.13	71.87 ± 1.24	77.44 ± 1.39	<0.0001	0.0084	0.0605	<0.0001^d^
LVM (g)^a^	1.17 (1.07,1.48)	1.62 (1.40,1.70)	1.40 (1.26,1.53)	0.0007	0.0494	0.0305	0.0015^b^
LVIDd (mm)^a^	6.57 (5.77,7.69)	8.55 (7.53,9.02)	7.77 (7.44,8.34)	0.0003	0.3933	0.0116	0.0011^b^

MAP, mean arterial pressure; HR, heart rate; LVEF, left ventricular ejection fraction; LVM, left ventricular mass; LVIDd, Left ventricular internal diameter end diastole.

a Median (25th percentile, 75th percentile).

b Kruskal-Wallis test with subsequent pairwise Mann-Whitney U tests.

c Mean (± standard error of the mean).

d ANOVA test with subsequent pairwise *t*-tests.

#### 3.1.2 Effects of Insulin Therapy on Measured Cardiovascular Parameters

The mean arterial pressure was not significantly different across the three groups. However, the mean heart rate decreased with diabetes, and declined even further with insulin treatment (Table 1). The left ventricular ejection fraction (LVEF) decreased among UDM, but it was rescued back to baseline among IDM (Table 1). Untreated diabetics experienced an increase in LV mass, which was partially rescued with insulin treatment (Table 1). The left ventricular internal diameter end diastole increased with diabetes, and insulin treatment did not reverse the diabetes-associated change (Table 1).

#### 3.1.3 Effects of Insulin Therapy on Rat Myocardial Interstitial Fibrosis and Cardiomyocyte Apoptosis

**FIGURE 2:**
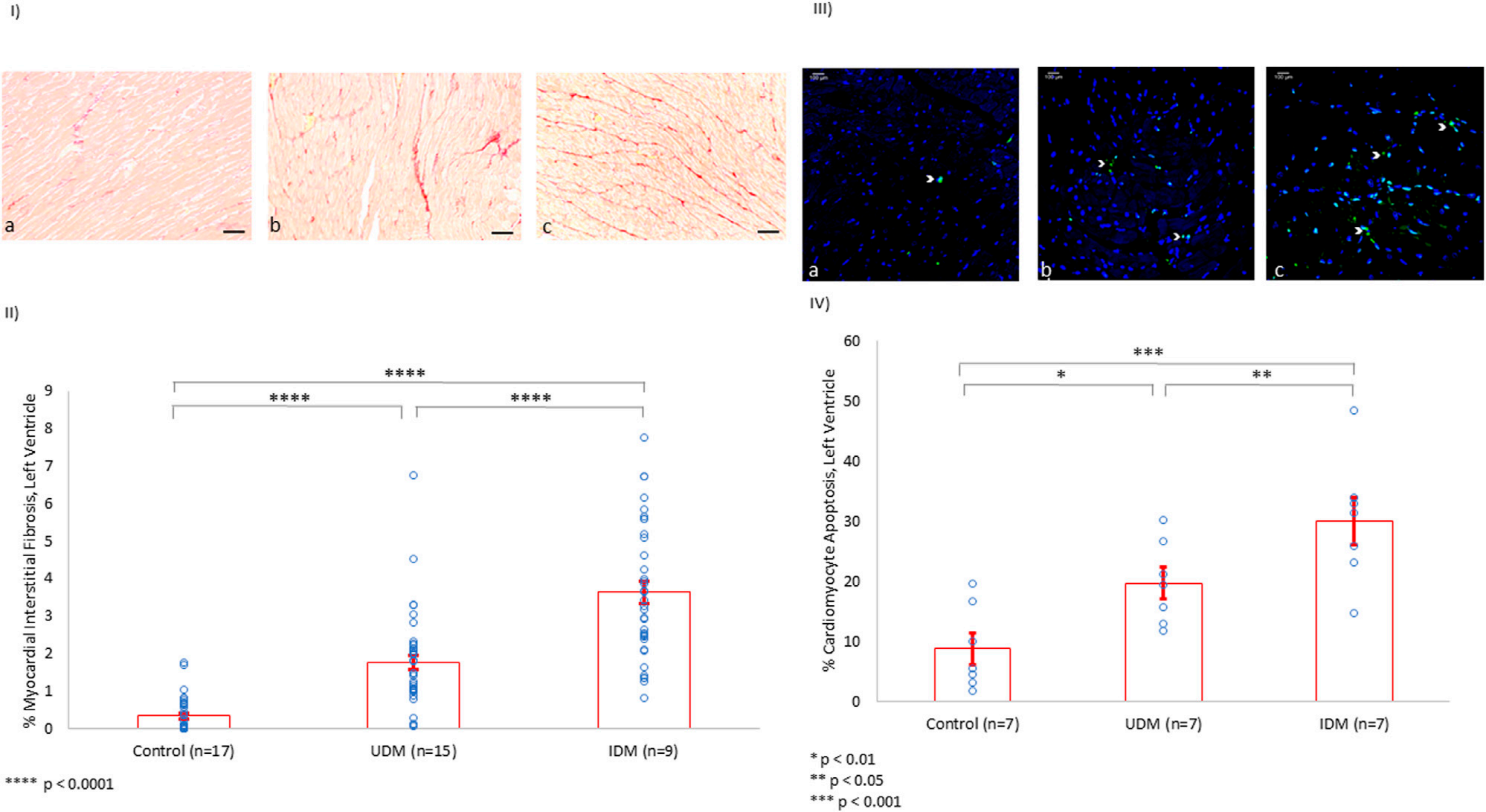
Left Ventricle Myocardial Interstitial Fibrosis and Cardiomyocyte Apoptosis (%). **(I)** Using Picrosirius red staining of collagen in the extracellular matrix, baseline LV fibrosis among controls (a) was measured (scale bar: 50 µm). UDM and IDM rats experienced a 5-fold (b) and 10-fold (c) increase in LV myocardial interstitial fibrosis, respectively. **(II)** Quantification of the myocardial interstitial fibrosis. **(III)** Using TUNEL assay, apoptotic material appeared green (white arrowhead). Compared to controls (a), the rate of apoptosis doubled in the left ventricle of untreated diabetic rats (b) and tripled among IDM rats (c). **(IV)** Quantification of the cardiomyocyte apoptosis in the LV (%).

Rat myocardial interstitial fibrosis was evaluated using Picrosirius red staining, and it was noted that UDM rats experienced more than a 5-fold increase in extracellular collagen deposition compared to controls. Furthermore, insulin therapy doubled the extent of fibrosis compared to UDM (*p* < 0.0001) (Figures 2I,II).

Further, cardiomyocyte apoptosis was investigated using the TUNEL assay. Indeed, rat cardiomyocyte apoptosis doubled from baseline in UDM, and tripled with insulin therapy (*p* < 0.05) (Figures 2 III,IV)

### 3.2 *In Vitro* Studies

#### 3.2.1 Effects of Insulin on Human Cardiomyocyte AC16 Apoptosis and Human Cardiac Fibroblast α-SMA Expression

**FIGURE 3:**
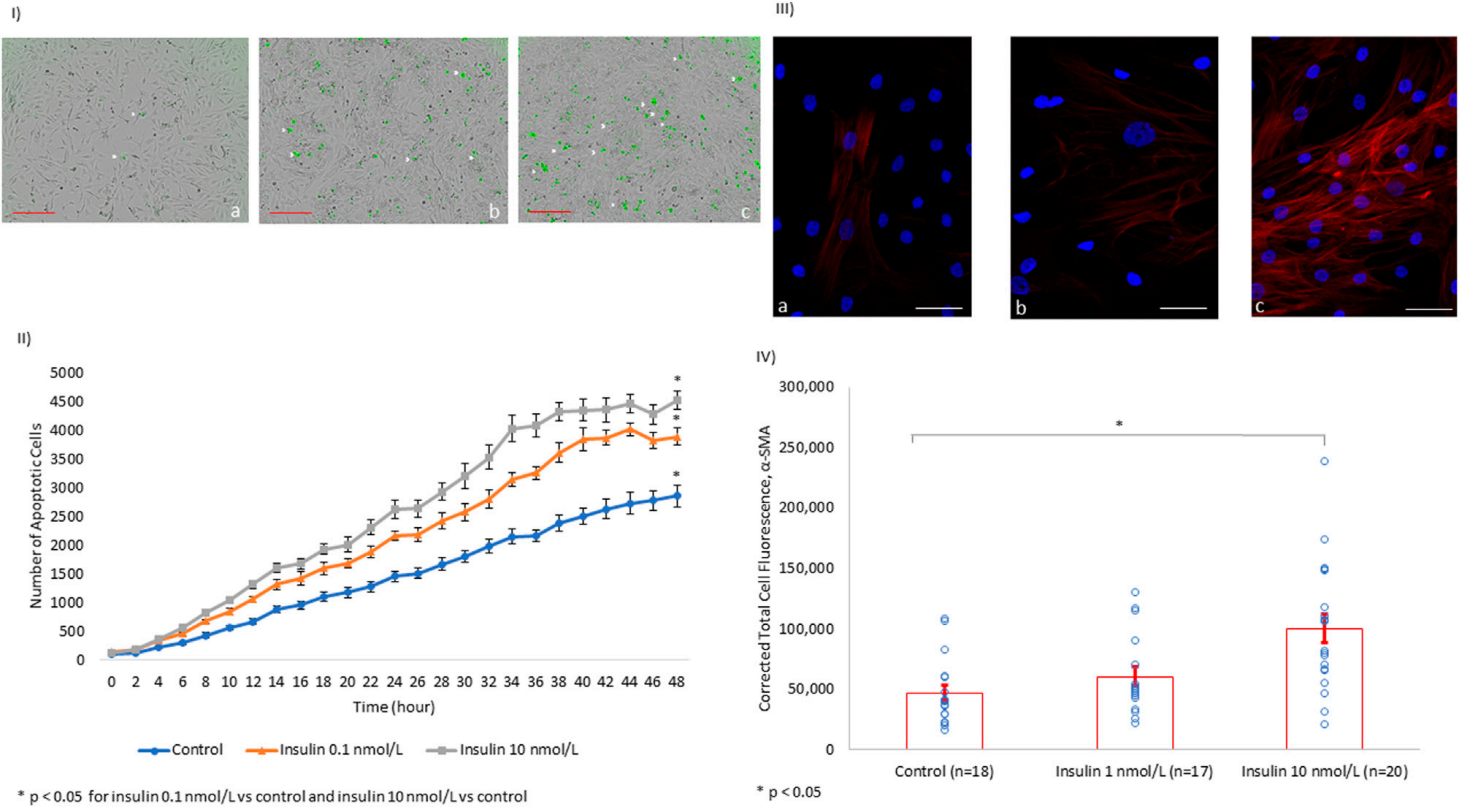
*In vitro*, Insulin Induces Cardiomyocyte Apoptosis and Myofibroblast α-SMA Expression in a Dose-dependent Manner. **(I)** Images of human cardiomyocytes (AC16) after 48 h of incubation with tissue culture media alone (a), tissue culture media + insulin 0.1 nmol/L (b), and tissue culture media + insulin 10 nmol/L (c). The number of apoptotic cells (white arrowhead) increases with the increasing concentration of insulin (scale bar: 300 µm). **(II)** Line chart quantifying the trend of apoptosis in human cardiomyocyte AC16 cultures over 48 h under different insulin concentrations. **(III)** HCF are shown with the nucleus (blue) and α-SMA (red) at baseline among controls (a). There is no significant difference in α-SMA expression in HFC treated with insulin at 1 nmol/L (b). However, the levels of α-SMA almost doubled with insulin treatment at 10 nmol/L (c, scale bar: 50 µm). **(IV)** Quantification of the α-SMA expression level using immunofluorescence.

To further explore the properties of insulin-associated apoptosis and fibrosis in the myocardium, we performed *in vitro* experiments on tissue cultures of human cardiomyocytes and human cardiac fibroblasts. We were able to show that insulin triggered apoptosis in AC16 cells in a dose-dependent manner (Figures 3I,II).

Furthermore, we evaluated fibrosis in HCF through quantifying alpha-smooth muscle actin (α-SMA) protein expression. α-SMA is used as a biomarker of fibroblast transformation into a stimulated fibrinogenic cell (i.e., a myofibroblast). We showed that insulin induced α-SMA overexpression in HCF in a dose-dependent manner (Figures 3III,IV).

#### 3.2.2 Comparing the Effects of Insulin With TGFβ1 on Human Cardiac Fibroblasts

**FIGURE 4:**
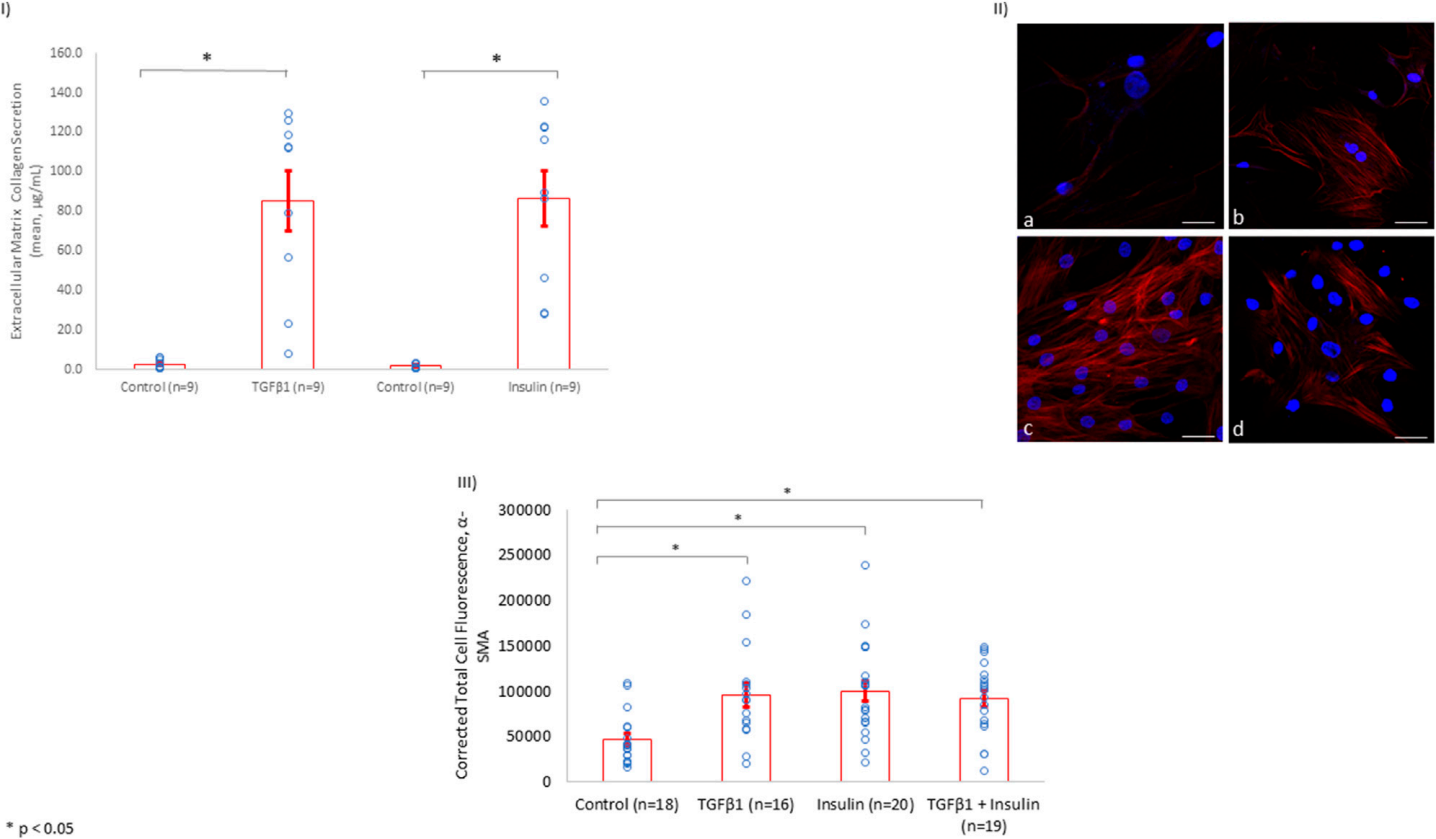
Insulin Induces Collagen Secretion and α-SMA Expression to Levels Similar to TGFβ1 in HCF. **(I)** Similar to the findings in rat LV tissue, insulin treatment (10 nmol/L) induced extracellular collagen secretion to a comparable degree with the known fibrosis-inducing agent, TGFβ1 (5 ng/ml). **(II)** Using total cell immunofluorescence, α-SMA levels were inspected among control HCF simply incubated in culture media for 72 h (a). α-SMA levels were upregulated after HCF incubation with TGFβ1 (5 ng/ml) (b), insulin (10 nmol/L) (c), or insulin (10 nmol/L) + TGFβ1 (5 ng/ml) (d) for 72 h in culture media (Scale bar: 50 µm). **(III)** Quantification of the of α-SMA expression levels using immunofluorescence in HCF among controls, HCF with TGFβ1, HCF with insulin, and HCF with insulin + TGFβ1.

Since fibrosis is associated with collagen secretion from fibroblasts, we investigated whether insulin and/or TGFβ1 incubation with HCF increased collagen secretion into the tissue culture media. We showed that insulin (10 nmol/L) significantly increased extracellular collagen secretion by HCF in a fashion similar to what was observed among HCF incubated with TGFβ1 (5 ng/ml) (Figure 4I).

In addition, after incubating HCF with insulin for 72 h, α-SMA was upregulated to a level similar to that observed when HCF were incubated with the fibrogenesis-mediator TGFβ1 (Figures 4II,III). Of note, incubating HCF with both insulin and TGFβ1 simultaneously did not change the level of α-SMA expression observed with either incubation alone, suggesting that insulin as well as TGFβ1 may share the same mechanism(s) for promoting fibrosis.

#### 3.2.3 SMAD2 Expression in Human Cardiac Fibroblasts

**FIGURE 5:**
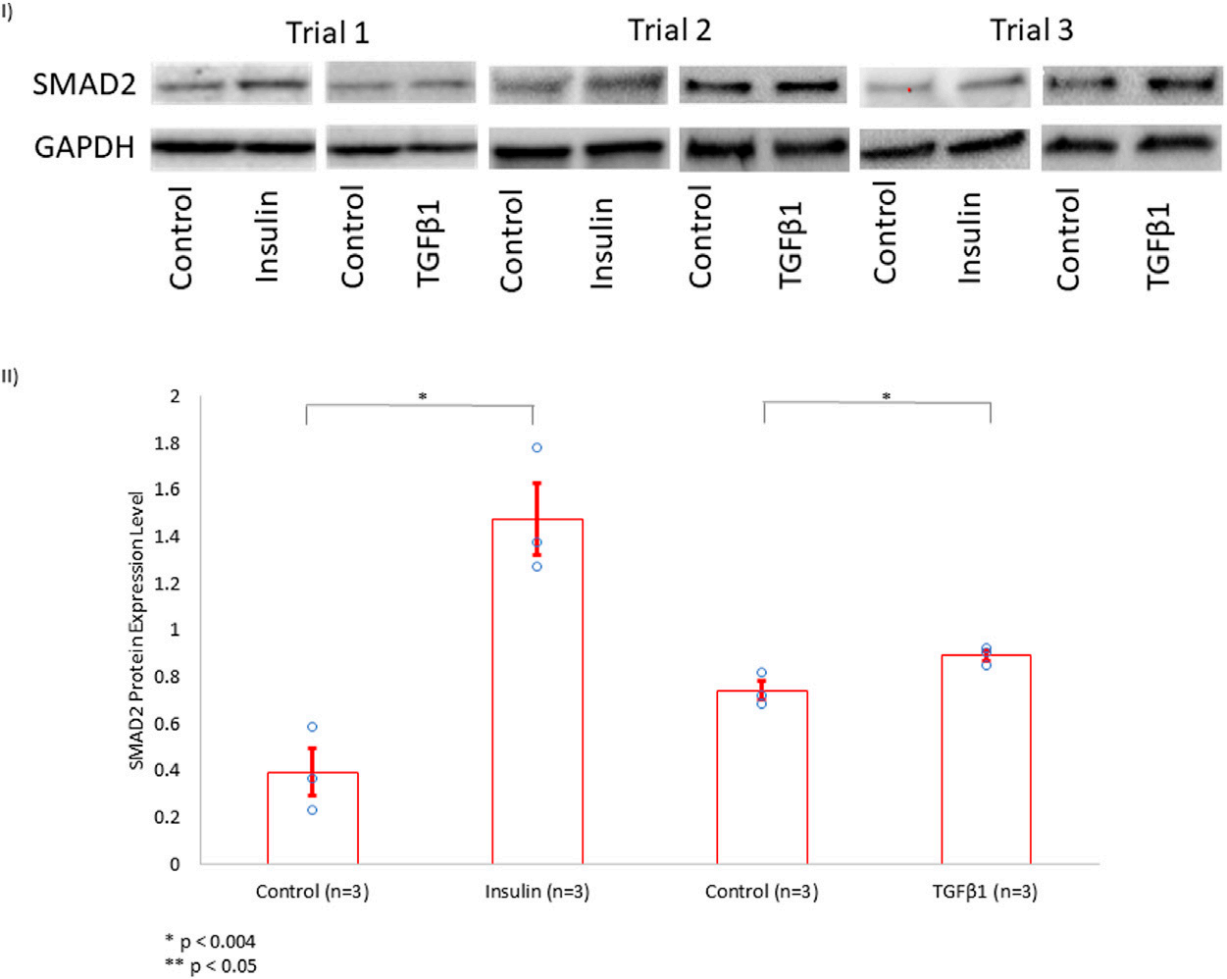
SMAD2 Protein Expression Level in HCF Treated with Insulin or TGFβ1. **(I)** In the presence of growth media, HCF were treated separately with control solution, insulin (10 nmol/L), or TGFβ1 (5 nmol/L) for 72 h. Subsequently, SMAD2 protein expression level was assessed using Western blotting, with GAPDH serving as a control for normalization. These experiments were done in triplicates (Trials 1, 2, and 3). **(II)** Band intensities were quantified using ImageJ software. Insulin increased SMAD2 protein expression level to more than 3.5 × when compared to controls. TGFβ1, serving as a positive control, also increased SMAD2 expression.

We showed that the incubation of HCF with both TGFβ1 and insulin resulted in similar collagen secretion and α-SMA expression compared to incubation with either agent alone (Figure 4). This finding suggested that insulin and TGFβ1 may induce fibrosis through a shared pathway. Since it is well-known that TGFβ1 induces fibrosis in a SMAD2-dependent pathway, we investigated whether insulin upregulated SMAD2 expression. Indeed, we found that insulin, similar to TGFβ1, significantly increased SMAD2 protein expression (Figure 5).

## 4 Discussion

In an experimental rodent model of DM, insulin exacerbated the DM-associated myocardial interstitial fibrosis and cardiomyocyte apoptosis. Supporting the *in vivo* findings, *in vitro*, insulin promoted human cardiomyocyte apoptosis and human cardiac fibroblast α-SMA expression in a dose-dependent manner. Further, in a fashion similar to the classical TGFβ1 fibrosis pathway, we showed that insulin increased the secretion of extracellular collagen and upregulated the intracellular expression of α-SMA and SMAD2. Collectively, and for the first time, our experimental findings support the hypothesis that insulin exerts direct profibrotic and proapoptotic effects on the left ventricle in diabetes; these findings could potentially explain some of the LV dysfunction, and therefore, worse cardiovascular outcomes experienced by insulin-utilizing diabetic patients. A longer study is warranted in the future to investigate the long-term effects of insulin on myocardial structure and function in diabetes.

Diabetes is associated with a 1.9-fold increased risk for the development of left ventricular dysfunction (Dandamudi et al., 2014), and diabetics with pre-clinical diastolic dysfunction have a significantly higher risk for the development of overt clinical heart failure and for mortality (From et al., 2010). Intriguingly, intensive glycemic control is associated with a higher incidence of CHF development (Nichols et al., 2004) and increased mortality without significantly improving major cardiovascular outcomes (Action to Control Cardiovascular Risk, 2008). Specifically, insulin emerged as an independent risk factor for prevalent and incident CHF (Nichols et al., 2004; Cosmi et al., 2018), with insulin-treated diabetes portending even worse mortality rates than diabetes alone (Smooke et al., 2005; Damluji et al., 2017; Cosmi et al., 2018).

Insulin is used in approximately one-third of patients with diabetes (Trief et al., 2016), and it has emerged as an independent risk factor for left ventricular dysfunction (Hirose et al., 2021) and heart failure hospitalizations (Nichols et al., 2004; Wang et al., 2015). In addition to insulin causing sodium retention and being indicative of a more advanced diabetic state (Cosmi et al., 2018), our findings suggest that it can inflict direct adverse changes to the left ventricular tissue. These changes may add to our understanding of why insulin-dependent diabetics experience worse cardiovascular outcomes (Smooke et al., 2005; Damluji et al., 2017; Cosmi et al., 2018). Our findings also suggest that perhaps the mechanism(s) through which insulin causes adverse cardiovascular effects are more complex than previously believed.

Next steps include investigating whether insulin-dependent diabetic patients truly experience worse left ventricular fibrosis and apoptosis than their non-insulin-dependent counterparts. In fact, if these findings are replicated in humans, then exploring strategies to block the excessive insulin signaling on the myocardium will need to be undertaken. Additionally, if our findings are replicable in humans, clinicians may have to exhaust all options before considering prescribing insulin for type 2 diabetic patients.

Alternatively, combining insulin therapy with cardioprotective antidiabetic medications, such as glucagon-like peptide 1 (GLP-1) agonists and sodium-glucose cotransporter-2 inhibitors (SGLT2i), may be another reasonable approach to protect against the unintended insulin-associated myocardial tissue damage. The latter strategy seems feasible, especially since one-sixth of type 2 diabetics use insulin in addition to another hypoglycemic agent (Trief et al., 2016). In fact, several GLP-1 agonists have already been shown to reduce adverse cardiovascular outcomes among diabetics (Sheahan et al., 2020). SGLT2i, such as Empagliflozin (Anker et al., 2021) and Dapagliflozin (McMurray et al., 2019), bestow favorable cardiovascular effects, regardless of the patients’ diabetic status (McMurray et al., 2019; Anker et al., 2021; Razuk, 2022).

In the current investigation, UDMs experienced significant weight loss, which may be attributed to lipolysis and muscle wasting in the setting of inadequate circulating plasma insulin (Dimitriadis et al., 2011). As anticipated, insulin therapy did partially rescue against the insulin deficiency-induced weight loss (Quinn et al., 2006; Dimitriadis et al., 2011). Consistent with prior findings (Bugger and Abel, 2014), diabetes was associated with increased LV mass, increased myocardial interstitial fibrosis, and increased cardiomyocyte apoptosis as compared to non-diabetic rats. Several mechanisms have been proposed for the increased fibrosis associated with DM, including upregulation of TGFβ1 signaling and dysregulation of matrix metalloproteinases (Bugger and Abel, 2014). Further, necrosis and apoptosis have been reported in diabetic hearts in humans and rodent models, which are associated with increased TGFβ1 signaling, renin–angiotensin–aldosterone system activation, and increased reactive oxygen species (Bugger and Abel, 2014). More importantly, in addition to confirming the previous findings, we first report that insulin therapy further increased the DM-associated myocardial interstitial fibrosis and cardiomyocyte apoptosis. The profibrotic properties of insulin have been demonstrated in adipose tissue (Hasegawa et al., 2018) and in stellate cells in both the liver (Cai et al., 2017) and the pancreas (Yang et al., 2016). However, our study provides the first reported evidence that insulin is profibrotic in cardiac tissue in a diabetic rat model. Additionally, the fact that insulin upregulated SMAD2 in myofibroblasts suggests that, similar to TGFβ1 (Derynck and Zhang, 2003), insulin may use a SMAD2-dependent pathway to induce fibrosis. Indeed, incubating cells with both insulin and TGFβ1 did not result in further α-SMA protein expression than when either agent is added separately, suggesting the possibility that both profibrotic agents may potentially share a similar cascade. The profibrotic properties of insulin may contribute to the understanding of why diabetics have worse clinical outcomes with insulin use.

Furthermore, we demonstrated that insulin induced cardiomyocyte apoptosis, both *in vitro* and *in vivo*. This contrasts with prior reports indicating that insulin can have anti-apoptotic effects via activating the PI3K/Akt pathway (Iliadis et al., 2011; Xing et al., 2015). One hypothesis may be that, in states of hyperinsulinemia, such as DM2 and being on an insulin pump, excess insulin signaling can be cytotoxic to cardiomyocytes, as is demonstrated by the dose-response curve in our *in vitro* studies. Indeed, acting through the insulin-receptor, insulin potentiated caspase-3-mediated apoptosis in beta cells exposed to high doses of insulin for a prolonged period of time (Guillen et al., 2008). Future studies that investigate the expression levels of proapoptotic proteins in cardiomyocytes when incubated with insulin are necessary to further delineate the mechanism(s) involved. Regardless, our study is the first to demonstrate the proapoptotic properties of insulin on cardiomyocytes in diabetes, and that may also account for the worsened clinical outcomes among insulin-utilizing diabetic patients.

Of note, despite the profibrotic effects of insulin on the myocardial tissue, the left ventricular ejection fraction remained intact. One explanation may be that myocardial fibrosis and cardiomyocyte apoptosis could have preceded measurable systolic dysfunction. Therefore, a longer treatment period with insulin in a diabetic rat model is certainly warranted to investigate the aforementioned hypothesis.

### 4.1 Limitations

'The rat model used in our study is an experimental diabetic model, which may limit the generalizability of our findings to humans. Further, insulin-delivering pumps were installed in IDM after having had DM for 2 months, and the treatment was only for 1 month. Additionally, the duration of insulin treatment for 1 month is relatively short, which may limit our understanding of the full, long-term effects of insulin on the myocardium. Moreover, diastolic dysfunction, which is expected to ensue with left ventricular fibrosis, was not assessed in this study, and it certainly needs to be addressed in a future set of experiments.

### 4.2 Conclusion

Through a series of *in vivo* and *in vitro* experiments, we elucidated that insulin therapy, while restoring euglycemia, was associated with increased myocardial interstitial fibrosis and increased cardiomyocyte apoptosis. Longer studies are warranted to characterize the long-term effects of insulin on cardiac structure and function.

## Data Availability

The raw data supporting the conclusions of this article will be made available by the authors, without undue reservation.

## Abbreviations

α-SMA, Alpha smooth muscle actin; AC16, Human cardiomyocyte cell line; CHF, Congestive heart failure; DM, Diabetes mellitus; GAPDH, Glyceraldehyde-3-Phosphate Dehydrogenase; GLP-1, Glucagon-like Peptide 1; HCF, Human Cardiac Fibroblasts; HR, Heart rate; IDM, insulin-treated diabetic rats; LV, left ventricle; LVEF, Left ventricular ejection fraction; LVM, Left ventricular mass; LVIDd, Left ventricular internal diameter end diastole; MAP, Mean arterial pressure; SMAD2, Mothers against decapentaplegic gene family 2; SGLT2i, Sodium-Glucose Cotransporter-2 inhibitors; TGFβ1, Transforming Growth Factor Beta 1; UDM, untreated diabetic rats.
